# A validated method for analysis of Swerchirin in *Swertia longifolia* Boiss. by high performance liquid chromatography

**DOI:** 10.4103/0973-1296.59961

**Published:** 2010-02-13

**Authors:** M. Shekarchi, H. Hajimehdipoor, M. Khanavi, N. Adib, M. Bozorgi, B. Akbari-Adergani

**Affiliations:** *Food and Drug Control Laboratories and Food and Drug Laboratory Research Center, MOH and ME, Tehran, Iran*; 1 *Department of Pharmacognosy, Faculty of Pharmacy, Tehran University of Medical Sciences, Tehran, Iran*; 2 *Pharmaceutical Sciences Branch, Azad University, Tehran, Iran*

**Keywords:** High performance liquid chromatography, Swerchirin, *Swertia longifolia*, validation

## Abstract

*Swertia* spp. (Gentianaceae) grow widely in the eastern and southern Asian countries and are used as traditional medicine for gastrointestinal disorders. Swerchirin, one of the xanthones in *Swertia* spp., has many pharmacological properties, such as, antimalarial, antihepatotoxic, and hypoglycemic effects. Because of the pharmacological importance of Swerchirin in this investigation, it was purified from *Swertia longifolia* Boiss. as one of the main components and quantified by means of a validated high performance liquid chromatography (HPLC) technique. Aerial parts of the plant were extracted with acetone 80%. Phenolic and non-phenolic constituents of the extract were separated from each other during several processes. The phenolic fraction was injected into the semi-preparative HPLC system, which consisted of a C_18_ column and a gradient methanol: 0.1% formic acid mode. Using this method, we were able to purify six xanthones from the plant, in order to use them as standard materials. The analytical method was validated for Swerchirin as one of the most important components of the plant, with more pharmacological activities according to the validation parameters, such as, selectivity, linearity (*r*^2^ > 0.9998), precision (≤3.3), and accuracy, which were measured by the determination of recovery (98-107%). The limits of detection and quantization were found to be 2.1 and 6.3 μg/mL, respectively. On account of the speed and accuracy, the UV-HPLC method may be used for quantitative analysis of Swerchirin.

## INTRODUCTION

**Figure 1:**
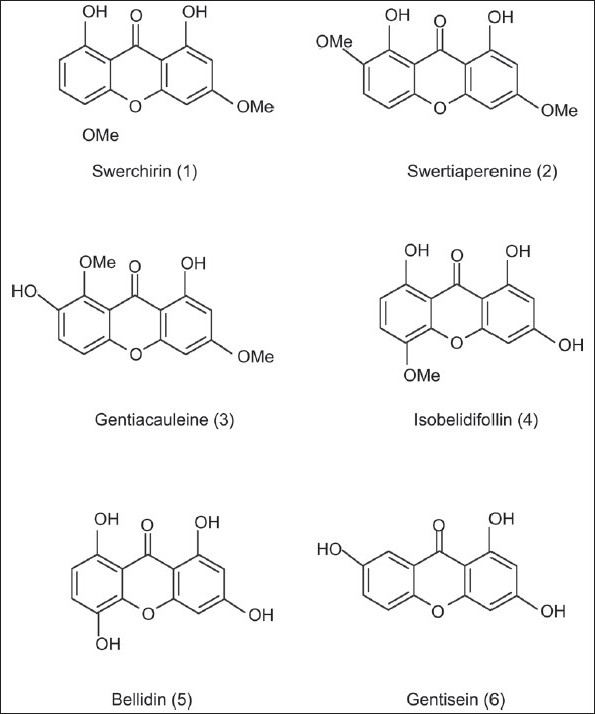
Chemical structures of xanthones isolated from *Swertia longifolia*

Plants of genus *Swertia* (Gentianaceae) are widely distributed in the eastern and southern Asian countries, mainly in Japan, China, India, and Pakistan. These plants have been used in traditional medicine for treatment of hepatic, choleric, and inflammatory diseases.[1] Despite different components in the *Swertia* species, xanthones are the most prominent naturally occurring compounds in this genus, which contain a distinctive polyphenolic structure and show many pharmacological effects, such as, antioxidant,[2] antimalarial,[3] antibacterial,[4] antitumor,[5] anti-diabetes,[6,7] and hepatoprotective properties.[8] There are many separation methods that are able to isolate xanthones via exhaustive extraction and manipulation schemes. Due to species, geographic, climatic, and environmental factors, several types of xanthones have been isolated from *Swertia spp*.[9,10] Several methods including high performance liquid chromatography (HPLC),[11–14] thin layer chromatography (TLC),[15] and capillary electrophoresis,[16] have been reported for the analysis of different classes of xanthones, but few quantitative analytical methods have been reported for determining xanthones in the *Swertia* species. In accordance with our previous studies on phytochemistry of *Swertia longifolia* Boiss. (an endemic plant of Flora Iranica),[17,18] six xanthones Swerchirin (1), Swertiaperenine (2), Gentiacauleine (3), Isobillidifolin (4), Bellidin (5), and Gentisein (6), with different structures, have been separated and their structures elucidated [Figure 1]. Our investigations reveal that xanthones isolated from the Iranian genus are mostly different from those derived in Japan and China. As the application of *Swertia longifolia* as a medicinal plant has been extended, a reliable standard method is required to determine its quality. Swerchirin, which is one of the major components in the plant, shows pharmacological properties such as antimalarial, antihepatotoxic, and antidiabetic effects,[1,8] therefore, for the first time, it has been targeted to be purified and quantified in this endemic plant. It is necessary to mention that Swerchirin by itself cannot be responsible for all pharmacological effects of *Swertia longifolia*. It is obvious that the synergic effects of all compounds in the plant play a significant role in the pharmacological properties. In this investigation, xanthones 1-6 were purified using semi-preparative HPLC, and Swerchirin (1), as one of the main components in the plant, was analyzed by using the validated UV-HPLC method.

## MATERIALS AND METHODS

### Plant material

The aerial parts of *Swertia longifolia* were collected in June 2007 from the North of Iran, Mazandaran province, Lavashm mountains, approximately 2900 m, H. Hajimehdipoor and were identified by Dr. V. Mozaffarian, Botanist from the Research Institute of Forests and Rangelands (Tehran).

### Instrumentation

A preparative HPLC experiment was performed using the Knauer system equipped with a vacuum degasser, quaternary solvent mixer, and K-2600 UV-detector. The preparative column Merck Lichrocart 100 RP-18 end-capped (10 × 250 mm, 10 μm) provided excellent separations when 0.5 mL volumes of the extract were injected and a gradient of 60-90% methanol-formic acid 0.1% was extended to 30 minutes, at the flow rate of 5 mL/minute. The analytical HPLC experiment was performed using a Waters Alliance system equipped with a vacuum degasser, quaternary solvent mixer, auto-sampler, and a waters 2996 diode array detector. The UV spectra were collected across the range of 200-900 nm, extracting 254 nm for chromatograms. Empower software was utilized for instrument control, data collection, and data processing. The column was an ACE C_18_ (4.6 × 250 mm, 5 μm). The mobile phase and programming were the same as preparative chromatography. The flow rate was 1 mL/minute. The injection volume for all samples and standards was 20 μL.

### Chemicals

All solvents were obtained from Merck Co. (Darmstadt, Germany). Water used in all the experiments was deionized by Purelab UHQ Elga. Standard compounds 1-6 were isolated in our laboratory by mixing 1 kg of dried and milled aerial parts of the plant with 5L 80:20 acetone-water at 60°C for eight hours. The extract was concentrated under reduced pressure. A solution of 0.1 N sodium hydroxide (200 mL) was added and after shaking, the mixture was filtered and adjusted to acidic pH by hydrochloric acid (1 N). The acidic solution was extracted by dichloromethane (3 × 250 mL) and the combined extracts were dried under reduced pressure. The resulting solids were dissolved in acetonitrile and purified by semi-preparative HPLC. Fractions from multiple injections were collected with an automatic fraction collector and pooled. The individual xanthone solids were recovered under reduced pressure and recrystallized from ethanol-water solutions. The structures of these compounds were identified by comparing the^1^ H-NMR,^13^ C-NMR, mass, and UV spectral data with those reported,[17,18] and the purity of each compound was checked by HPLC.

### Determination of Swerchirin content

#### Optimization of the extraction procedure

*Solvent effect:* The samples were prepared in hexane, dichloromethane, acetone, and acetone-water 80:20 to determine the effect of the solvent on the extraction efficiency.

*Optimization of the sample size*: To evaluate the effect of the sample size on the accuracy of the Swerchirin content estimation, the samples were prepared in triplicate in two sets. In the first set, 0.1 g and in second set 0.5 g of powder were weighted and used for extraction.

*Effect of the extraction method*: To determine the effect of the procedure on the extraction, hot solvent extraction, maceration, and sonification were compared.

#### Sample preparation for analytical high performance liquid chromatography

To prepare samples for HPLC analysis, 0.1 g of dried and milled aerial parts of *Swertia longifolia* were placed in a 50 mL glass tube and extracted with 30 mL hexane for one hour at 40°C. The suspension was filtered and the remaining powder was extracted two more times using 30 mL hexane. The filtrate was concentrated under reduced pressure, dissolved in 25 mL acetonitrile, and filtered through a 0.45μm PTFE filter, and 20 μL of the final solution was injected into the HPLC system.

#### Preparation of standard solutions

A standard solution of Swerchirin was prepared by dissolving 10 mg of the compound in 10 mL of acetonitrile. Serial dilutions were made in the range of 6-24 μg/mL, to plot the calibration curve.

### Validation

The reliability of the HPLC-method for analysis of Swerchirin was validated through its linearity, selectivity, precision, and recovery.

#### Selectivity

For the chromatographic method, developing a separation involves demonstrating specificity, which is the ability of the method to accurately measure the analyte response in the presence of all interferences. Therefore, the extraction mixtures obtained from the sample preparation were analyzed and the analyte peak (Swerchirin) was evaluated for peak purity and resolution from the nearest eluting peak (Swertiaperenine).

#### Linearity

Due to the verification of the normal distribution of results, linearity was evaluated through the relationship between the concentration of Swerchirin and the absorbance obtained from the UV-HPLC detector. The determination coefficient (*r*^2^) was calculated by means of the least-square analysis.[19] The calibration line was achieved through two replicates of each concentration of Swerchirin (6-24 μg/ mL), to identify the extent of the total variability of the response that could be explained by the linear regression model.

#### Precision

The precision of each method indicates the degree of dispersion within a series on the determination of the same sample. Six real samples were analyzed on the same day (intra-day) and three on consecutive days (inter-day), and then the relative standard deviations were calculated. Each sample was injected to HPLC thrice.

#### Recovery

This parameter showed the proximity between the experimental values and the real ones. It ensured that no loss or uptake occurred during the process. The determination of this parameter was performed during the method by studying the recovery after a standard addition procedure, with two additional levels. Three replicate amounts of plant powder (0.3 g) were weighted and each of them was divided into three equal portions (0.1 g). One part was used as the real sample and others had been spiked with Swerchirin standard material (2 and 4 μg/mL). In each additional level, three determinations were carried out and the recovery percentage was calculated in every case. Each sample was injected into HPLC thrice.

## RESULTS AND DISCUSSION

### Extraction procedure

Naturally, xanthones are insoluble in water, but are soluble in a variety of other solvents, ranging from methanol to hexane.[15] An 80:20 acetone-water mixture was an excellent choice, and was selected from several mixtures of solvents for extraction of all xanthones from the plant, for purification purposes and preparation of standard materials. Ninety-eight to one hundred and seven percent recoveries obtained by the addition of 15 and 30% original Swerchirin into the sample matrix have shown that a hydrophobic solvent such as hexane was the best choice for the quantitative analysis of Swerchirin (the most hydrophobic compound). Besides the high recovery, it was less interfering in comparison with other solvents, making it a suitable solvent for extraction of Swerchirin. Hot solvent extraction was selected to compare maceration with sonification. Comparison between two experiments showed that the smaller sample size did not appear to have a significant effect on the accuracy of Swerchirin analysis. As large sample sizes required more solvents and space a small sample size was selected for the study. In general, hot extraction of the plant (0.1 g) with hexane for one hour (thrice) was selected as the best method for Swerchirin extraction.

### Method development and validation results

**Figure 2:**
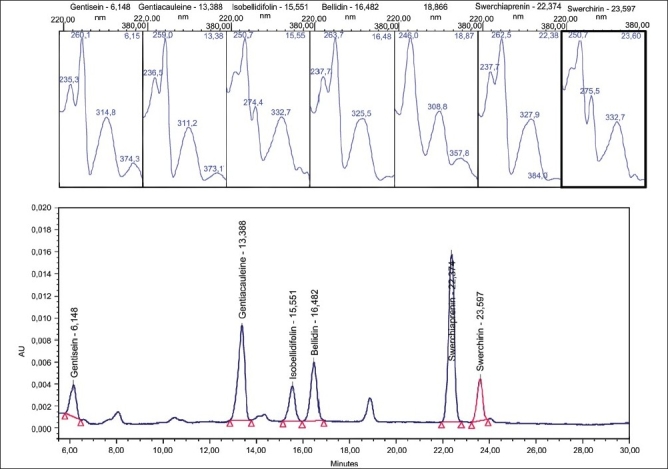
HPLC chromatogram of *Swertia longifolia* xanthones with characteristic UV spectra at 200-400 nm

**Figure 3:**
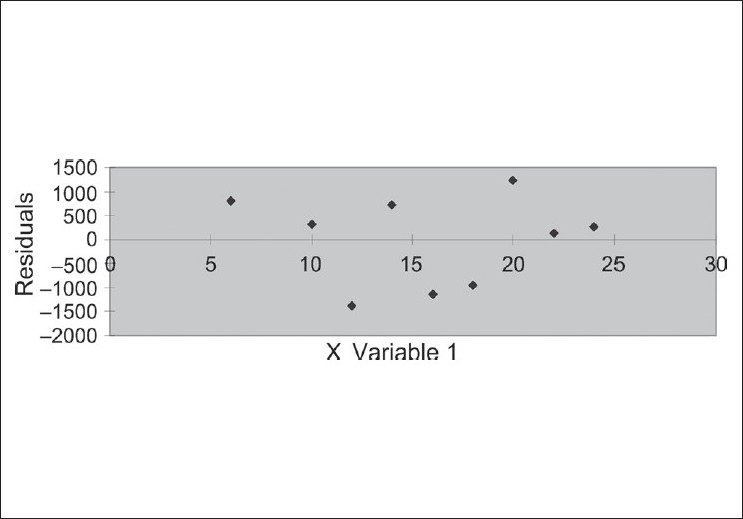
Residuals plotted against concentrations of Swerchirin in the calibration model

The two objectives of this study were to develop a sufficient semi-preparative HPLC method for the purification of the main components (especially Swerchirin) and to determine the amount of the targeted compound in *Swertia longifolia* Boiss. As is shown in [Figure 1], the structure of all the xanthones were di-, tri- or tetra-hydroxylated xanthones, with similar structures, especially in the case of Swerchirin and Swertiaperenine, which were structural isomers; therefore, it was difficult to separate all components simultaneously. After comparison between the different columns such as, C_8_, C_18_, CN, and phenyl, the best separation efficiency was obtained by using the C_18_ column. The mobile phase investigations showed that the ratio of organic modifiers, such as, the acetonitrile or methanol in the mobile phase, was the key to a good separation. The pH value played an important role in solute ionization. In order to minimize xanthones ionization, using an acidic mobile phase was obligated. According to this, the best separation was achieved by using 0.1% formic acid solution. The gradient mode of the instrument was changed to obtain the best resolution and the shortest run time. Each xanthone peak was resolved from the neighboring peaks and displayed excellent peak symmetry and separation efficiency as seen in Figure 2. These groups of compounds had a special chromophoric nature, which made them easy to identify from their UV diode-array absorption spectra. All the unique spectra had a strong, split absorption peak, in a maximum range of 230-300 nm. In addition, the secondary single peaks with lower extinction were observed at 310-370 nm [Figure 2]. Compounds 1-6 were isolated by preparative HPLC and recrystallized from the alcohol-water solution. The UV, ^1^HNMR, ^13^CNMR, and MS spectra of these isolated compounds were used to confirm them after comparison with literature.[17,18] The results obtained from the Swerchirin method validation according to linearity, selectivity, accuracy, and precision showed that the proposed method was suitable for the analysis of Swerchirin. Comparison between the purity threshold and purity angle reported in the empower software showed that the method was specific for Swerchirin and the reported peak was completely separated from the other interfering compounds. The linear relationship between the detector response and different concentrations of Swerchirin was confirmed in the range of 6-24 μg/mL, with a correlation coefficient of 0.99986 and equation y = 5308x + 2350. To ascertain the suitability of the linear calibration model, the residuals plotted versus concentrations showed a reasonable scatter of data [Figure 3]. Furthermore, the intercept corresponded with the acceptance limits (±2% of < Y > target value), hence, the linearity of the calibration model was confirmed. The overall limit of detection (LOD) and limit of quantitation (LOQ) were 2.1 and 6.3 μg/mL, respectively. To verify the precision and repeatability of the method, the results obtained from six repeated experiments on real samples, at a target concentration, on one day (intra-day) and on three consecutive days (inter-day) were analyzed. The relative standard deviations (RSDs%) of the intra-day and inter-day have been shown in Table 1. The results of intermediate precision using different analysts, different instruments, and on different days, showed that these parameters did not have any significant effect on the variation of results. After these validation studies, the method's ability to provide good quantization in our laboratory was confirmed. The last step in the measurement of precision (reproducibility), which focused more on the bias in results, rather than on determining the differences in precision alone, as inter-laboratory crossover studies, would be our next target. Accuracy, which was evaluated as recovery, after spiking the plant samples with standards at two concentration levels ranged between 98-107% [Table 2].

**Table 1:** Repeatability of Swerchirin analysis in *Swertia longifolia*

Days	Swerchirin content (μg/mL)						Mean ±SD (intra-day)	RSD% (intra-day)	RSD% (inter-day)
				
	A_1_	A_2_	A_3_	A_4_	A_5_	A_6_			
1	12.9	12.7	13.5	12.9	13.4	12.8	13.1 ± 0.3	2.7	
2	13.6	13.2	13.6	12.9	12.9	13.1	13.3 ± 0.3	2.4	3.3
3	14.0	13.8	13.9	13.8	13.5	14.4	13.9 ± 0.3	1.9	

**Table 2:** Recovery percent of Swerchirin added to *Swertia longifolia*

Added (μg/mL)	Found (μg/mL)	Recovery %	Mean recovery ± SD
0	13.6	-	-
	13.1	-	-
	13.3	-	-
2	15.5	107	
	15.3	99	102 ± 4.3
	15.3	100	
4	17.5	105	
	17.3	99	101 ± 3.7
	17.3	98	

## CONCLUSIONS

An improved semi-preparative HPLC method has been developed to separate and purify six xanthones, including Swerchirin (1), Swertiaperenine (2), Gentiacauleine (3), Isobillidifolin (4), Bellidin (5), and Gentisein (6) in *Swertia longifolia* Boiss. The good scale up and considerable resolution obtained in the preparative method gave us the opportunity to prepare pure and a sufficient amount of standard materials, especially for Swerchirin, which were not available as purified compounds for use in analytical procedures. The newly established analytical method was validated to be sensitive, precise, and accurate for the quantitative analysis of Swerchirin, the most active component in *Swertia longifolia* Boiss.

It can be concluded that this method is not only a useful tool for screening and determining more xanthones, but also an effective quality control method for the assay of Swerchirin in *Swertia longifolia*.
